# i6mA-DNCP: Computational Identification of DNA *N*^6^-Methyladenine Sites in the Rice Genome Using Optimized Dinucleotide-Based Features

**DOI:** 10.3390/genes10100828

**Published:** 2019-10-20

**Authors:** Liang Kong, Lichao Zhang

**Affiliations:** 1 School of Mathematics and Information Science & Technology, Hebei Normal University of Science & Technology, Qinhuangdao 066004, China; 2 School of Mathematics and Statistics, Northeastern University at Qinhuangdao, Qinhuangdao 066004, China; zhanglichaoouc@126.com; 3 College of Sciences, Northeastern University, Shenyang 110819, China

**Keywords:** *N*^6^-methyladenine, dinucleotide composition, DNA properties, bagging

## Abstract

DNA *N*^6^-methyladenine (6mA) plays an important role in regulating the gene expression of eukaryotes. Accurate identification of 6mA sites may assist in understanding genomic 6mA distributions and biological functions. Various experimental methods have been applied to detect 6mA sites in a genome-wide scope, but they are too time-consuming and expensive. Developing computational methods to rapidly identify 6mA sites is needed. In this paper, a new machine learning-based method, i6mA-DNCP, was proposed for identifying 6mA sites in the rice genome. Dinucleotide composition and dinucleotide-based DNA properties were first employed to represent DNA sequences. After a specially designed DNA property selection process, a bagging classifier was used to build the prediction model. The jackknife test on a benchmark dataset demonstrated that i6mA-DNCP could obtain 84.43% sensitivity, 88.86% specificity, 86.65% accuracy, a 0.734 Matthew’s correlation coefficient (MCC), and a 0.926 area under the receiver operating characteristic curve (AUC). Moreover, three independent datasets were established to assess the generalization ability of our method. Extensive experiments validated the effectiveness of i6mA-DNCP.

## 1. Introduction

*N*^6^-methyladenine (6mA), which results from the post-replicative modification of DNA by DNA methylases, has been found in both prokaryotes and eukaryotes, even though the rate of adenine methylation can differ greatly between species [1,2]. It used to be considered that 6mA functioned only in prokaryotes, where 6mA played an important role in discriminating the host DNA from foreign pathogenic DNA and protecting the host genome via the restriction-modification system [2,3]. However, the biological functions of 6mA in eukaryotes, especially higher eukaryotes, still remain largely unclear. Mapping and analyzing genomic 6mA distributions is fundamental for the elucidation of potential biological functions of DNA 6mA modification [1,4,5]. In recent studies [3,6,7,8,9,10,11,12,13,14], several high-throughput sequencing technologies were used to identify genomic distribution patterns of 6mA in higher eukaryotes, including green algae, worms, flies, frogs, pigs, mice, *Arabidopsis*, and rice. In worms, 6mA is broadly and evenly distributed across the genome [7], whereas 6mA is enriched at transposable elements in flies [8]. In frogs, 6mA is generally depleted from gene exons [9]. By contrast, 6mA is more frequently distributed in promoters and exons in the rice genome, and the 6mA genomic distributions are relatively conserved between *Arabidopsis* and rice [14]. Therefore, 6mA distribution patterns are rather species-specific in eukaryotes, which will lead to diverse functional roles.

To facilitate the characterization of 6mA distribution patterns and further functional analysis, genomic 6mA sites should be accurately identified at first. To this end, a number of experimental methods have been applied, such as liquid chromatography coupled with tandem mass spectrometry (LC-MS/MS) [15] and single-molecule real-time (SMRT) sequencing [16]. However, there are two problems existing in detecting 6mA sites by using these experimental methods. The first one is that there are some weaknesses in the current methods. For example, antibody detection is not quantitative and may be confounded by the recognition of other adenine base modifications, and the results of antibody detection and LC-MS/MS could be affected by bacterial contamination. The widely used SMRT sequencing cannot distinguish between 6mA and *N*^1^-Adenine (1mA) [2]. The second problem is that genome-scale detection of 6mA sites by biological assays is rather time-consuming and expensive. Thus, developing computational methods to rapidly identify 6mA sites is really needed. Motivated by this, a machine learning-based method named iDNA6mA-PseKNC was constructed to identify 6mA sites in the *Mus musculus* genome [17]. Subsequently, Chen et al. [18] proposed a computational method named i6mA-Pred to identify 6mA sites in the rice genome. I6mA-Pred uses nucleotide chemical properties and nucleotide frequency to encode DNA sequences. The overall accuracy of 83.13% was reported by the jackknife test on the benchmark dataset constructed by the authors. Recently, another two predictors (iDNA6mA [19] and iDNA6mA-Rice [20]) were further proposed to identify 6mA sites in the rice genome. IDNA6mA model is based on the deep learning approach. IDNA6mA-Rice model is based on random forest and mono-nucleotide binary encoding.

Considering the severe lack of a computational method in this field, we aimed to develop a new 6mA site prediction model to facilitate DNA 6mA modification analysis. In general, two key aspects should be considered in this prediction task. One is encoding DNA sequences with distinctive features. The other is selecting or designing a powerful classifier to train the prediction model. In this study, we encoded DNA sequences with dinucleotide composition and dinucleotide-based DNA properties (including 12 physical properties and three thermodynamic properties). To the best of our knowledge, this is the first time those features have been used to identify 6mA sites. To optimize feature space, a heuristic DNA property selection algorithm was designed. Then, five powerful classifiers (including three individual and two ensemble classifiers) were investigated, and the best-performing classifier was selected to build the final prediction model called i6mA-DNCP. Extensive assessments show that i6mA-DNCP outperforms the state-of-the-art methods. I6mA-DNCP is an effective and promising computational tool to identify DNA 6mA sites in the rice genome.

## 2. Materials and Methods

### 2.1. Dataset

A benchmark dataset was used to evaluate and compare the proposed method with existing methods. The dataset was acquired from http://lin-group.cn/server/i6mAPred/data. There are 1760 41-nt long DNA sequences, wherein 880 sequences containing 6mA sites are regarded as positive samples and 880 sequences contain non-6mA sites regarded as negative samples. We used this dataset for two reasons. On one hand, that dataset was the first and only public benchmark dataset for identifying 6mA sites in the rice genome. That enabled us to directly compare our results with other methods. On the other hand, the lower level of pairwise sequence identity (<60%) is rational to build a reliable prediction model. The details of how this dataset was constructed can be referred to in [18].

### 2.2. Feature Extraction

Each DNA sequence investigated in this study was 41 nt long, thus it can be represented as

R_1_R_2_…R_21_…R_41_, (1)

where the nucleotide at the center (i.e., R_21_) represents methylated or non-methylated adenine (A), and other nucleotides R*_i_* (*i*≠21) can be any one of the four bases (adenine (A), cytosine (C), guanine (G), and thymine (T)). By combining each pair of adjacent nucleotides, the dinucleotide sequence can be obtained and represented as

D_1_D_2_…D*_i_*…D_40_, (2)

where the order of a dinucleotide is defined by the order of the first nucleotide in the dinucleotide; i.e., D*_i_* = R*_i_*R*_i+1_* (*i* = 1, 2, …, 40). Based on the dinucleotide sequence, dinucleotide composition and dinucleotide-based DNA properties were used to represent DNA sequences.

#### 2.2.1. Dinucleotide Composition

Dinucleotide composition describes the occurrence frequencies of the 16 basic dinucleotide elements in a DNA sequence. It thus generates a 16-dimensional feature vector which is formulated as

(*f*(AA), *f*(AC), *f*(AG), *f*(AT),…, *f*(TT))^T^. (3)

Dinucleotide composition partially reflects the sequence order information and fragment information.

#### 2.2.2. Dinucleotide-Based DNA Properties

In order to convert a dinucleotide sequence into a numerical sequence with equal length, we used the DNA properties which can be represented by 16 numerical values with each value corresponding to a basic dinucleotide element. Given a specific property, the property profile (*p*_1_, *p*_2_, …, *p*_16_) can be constructed, where *p_i_* (*i* = 1, 2, …, 16) represents the numeric code of the *i*-th basic dinucleotide element. The property profile can be applied to construct a 40-dimensional feature vector

(*θ*_1_, *θ*_2_, …,*θ_i_*,…,*θ*_40_)^T^, (4)

where

(5) $$\theta_{i}=\{\begin{array}{l} p_{1},{\mathrm{where}\;D}_{i}\;\mathrm{is}\;\mathrm{the}\;1\mathrm{st}\;\mathrm{basic}\;\mathrm{dinucleotide}\;\mathrm{element}\\ p_{2},{\mathrm{where}\;D}_{i}\;\mathrm{is}\;\mathrm{the}\;2\mathrm{nd}\;\mathrm{basic}\;\mathrm{dinucleotide}\;\mathrm{element}\\ \cdots \cdots \\ p_{16},{\mathrm{where}\;D}_{i}\;\mathrm{is}\;\mathrm{the}\;16\mathrm{th}\;\mathrm{basic}\;\mathrm{dinucleotide}\;\mathrm{element} \end{array}.$$

In this study, 15 DNA properties from [21] were used. These properties can be divided into two groups. One group contains DNA physical properties including six kinds of dinucleotide flexibility parameters and six kinds of structural parameters. The other group contains DNA thermodynamic properties, including dinucleotide free energy, entropy, and enthalpy. We used these properties for two reasons. First, several studies have indicated that adenine methylation may have effects on DNA’s structure and/or the stability of the DNA structure [22]. Second, it is suggested that there is a relationship between 6mA distribution pattern in the rice genome and nucleosome positioning, and DNA flexibility also plays an important role in nucleosome positioning [14,23,24]. These DNA properties have been successfully applied in some DNA-related prediction problems, such as the identification of recombination spots [18,25,26,27] and DNase I hypersensitive sites [28,29,30]. We expect these properties could be effective for identifying 6mA sites.

**Figure 1:**
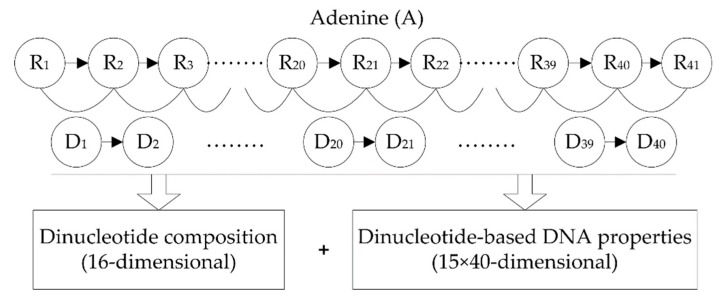
Feature extraction scheme for a given DNA sequence.

With the 15 DNA properties, 15 40-dimensional feature vectors were generated for each DNA sequence. We concatenated those feature vectors into a 600-dimensional feature vector. It should be pointed out that the original parameters of each DNA property were first normalized by the Z-score method, and then used to generate DNA features. The original and normalized parameters of the 15 DNA properties were listed in Supplementary Tables S1 and S2. As a result, we extracted 616 DNA features in total to represent a given DNA sequence by integrating dinucleotide composition and dinucleotide-based DNA property features. The proposed feature extraction scheme is shown in Figure 1.

### 2.3. DNA Property Selection

In general, a great number of DNA properties will result in more DNA features, thus redundancy information will be inevitable. To find out which properties are most suitable to identify 6mA sites, an exhaustive investigation of 2*^N^*-1 (*N* = 15) property sets is impractical. Hence, we designed a heuristic DNA property selection process to obtain a suboptimal property set. Given a universal set containing all the DNA properties, property selection began with an empty set. In each of the following iterations, the properties not yet selected were sequentially added into the set identified in the last iteration to generate a series of candidate sets. The performance of these candidate sets was evaluated by accuracy (see the definition in Section 2.5) based on a specific classifier and DNA features corresponding to the properties in the current candidate set. The candidate set with the highest accuracy was reserved and identified as the selected property set in the current iteration. This process repeated until all the properties had been selected or the highest accuracy of the current candidate sets was no better than the accuracy of property set identified in the last iteration. The pseudo-code of the above DNA property selection process is shown in Algorithm 1.

**Algorithm 1.** Heuristic DNA property selection.**Input:** Universal set $U=\{P_{1},P_{2},\ldots ,P_{N}\}$ **Output:** Optimized property set *S*1.  S←∅ 2.  Acc(S)←0     Acc(S) is the accuracy corresponding to *S* 3.  **while**
U\S≠∅ do 4.    **for** each property $P_{i_{k}}\in U\backslash S$ do 5.    generating a candidate set $S_{i_{k}}\leftarrow S\cup \{P_{i_{k}}\}$ 6.    calculating $\mathrm{Acc}(S_{i_{k}})$ 7.    **end for** 8.    $i*\leftarrow \underset{i_{k}}{\arg\max}\{\mathrm{Acc}(S_{i_{k}})\}$ 9.    **if**
$\mathrm{Acc}(S_{i*})\geq \mathrm{Acc}(S)$ do 10.   $S\leftarrow S_{i*}$ 11.   $\mathrm{Acc}(S)\leftarrow \mathrm{Acc}(S_{i*})$ 12.   **else** 13.   **break while** 14.   **end if** 15.   **end while** 16. **return**
*S*

**Figure 2:**
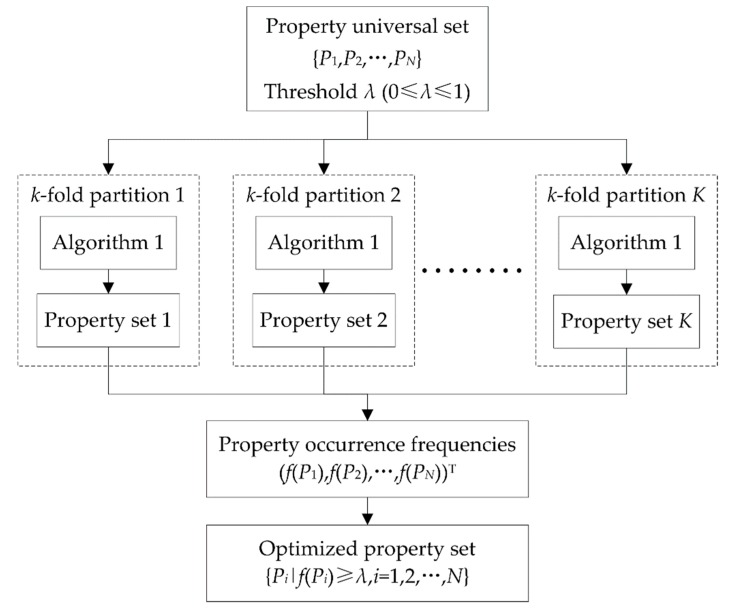
The workflow of DNA property selection.

To avoid overfitting, we calculated all the accuracies of property sets in Algorithm 1 by cross-validation. In statistical prediction, *k*-fold cross-validation and jackknife test or leave-one-out cross-validation is often used to evaluate the performance of a prediction model. Although a jackknife test can generate a unique partition on a dataset, it was not applicable here, due to its high computational load. By contrast, the general *k*-fold cross-validation is more efficient, and thus we used it to select properties. However, *k*-fold cross-validation partitions a dataset into *k* subsets randomly. It would result in the uncertainty of the selected properties. That is to say, different property sets would be obtained by implementing Algorithm 1 in different rounds. To partially offset the randomness from *k*-fold cross-validation and increase the reliability of the selected properties, we implemented identical *k*-fold cross-validations throughout the property selection process of Algorithm 1 in one round. Furthermore, we implemented Algorithm 1 more than once with different *k*-fold cross-validations, and synthesized all the selected property sets to identify the final, optimized property set. In detail, suppose that *K* independent property selection processes were implemented; then, we could obtain *K* different property sets and calculate the occurrence frequency of each property. The properties with occurrence frequencies no less than a given threshold *λ* (0≤*λ*≤1) formed the final optimized property set. The complete DNA property selection process is shown in Figure 2.

### 2.4. Classification Algorithms

Various machine-learning methods with varying abilities to learn categories have been successfully applied in computational genomics [31,32,33,34,35]. Considering the diversities of different machine-learning methods, we expected to choose a competent method to train the prediction model for identifying 6mA sites. In this study, Naive Bayes, logistic regression, support vector machine (SVM), LogitBoost, and bagging algorithms were investigated by contrast experiments. All these algorithms were used in MATLAB R2015b, where SVM with radial basis function (RBF) kernel was based on the publicly available software package LIBSVM [36] (https://www.csie.ntu.edu.tw/ ~cjlin/libsvm/) and the other four algorithms are based on the Statistics and Machine Learning Toolbox of MATLAB R2015b itself.

Since the prediction model is based on the bagging performed best (see Section 3.2 and Section 3.3), other algorithms are not mentioned in this section. Bagging, also named bootstrap aggregating, is a machine learning ensemble meta-algorithm designed to improve the stability and accuracy of individual machine learning algorithms used in statistical classification and regression. It is based on the idea of dividing the input dataset into a certain number of subsample datasets called bootstrap samples, building prediction model using a base learner on each bootstrap sample, and then aggregating these base models by voting scheme. Here we used classification and regression trees (CART) as a base learner, and thus denoted this algorithm as TreeBagging for convenience.

### 2.5. Performance Evaluation

In this study, multiple performance evaluation methods were used for assessing our prediction model. The 10-fold cross-validation was mainly applied to DNA property selection in the prediction model construction stage. The jackknife test was implemented to generate unique results for examining the model. Four metrics, including sensitivity (Sn), specificity (Sp), accuracy (Acc), and Matthew’s correlation coefficient (MCC) were used to quantify the prediction performance. They are defined as

(6) $$\mathrm{Sn}=\frac{\mathrm{TP}}{\mathrm{TP}+\mathrm{FN}},$$

(7) $$\mathrm{Sp}=\frac{\mathrm{TN}}{\mathrm{TN}+\mathrm{FP}},$$

(8) $$\mathrm{Acc}=\frac{\mathrm{TP}+\mathrm{TN}}{\mathrm{TP}+\mathrm{FN}+\mathrm{TN}+\mathrm{FP}},$$

(9) $$\mathrm{MCC}=\frac{\mathrm{TP}\times \mathrm{TN}-\mathrm{FP}\times \mathrm{FN}}{\sqrt{(\mathrm{FP}+\mathrm{TP})(\mathrm{TP}+\mathrm{FN})(\mathrm{TN}+\mathrm{FP})(\mathrm{TN}+\mathrm{FN})}},$$

where TP, TN, FP, and FN represent true positive, true negative, false positive, and false negative, respectively. In addition, we calculated the area under the receiver operating characteristic curve (AUC) to evaluate the prediction performance. Note that Acc, MCC, and AUC are three comprehensive metrics. A higher value of Acc, MCC, or AUC means better prediction performance of a prediction model.

## 3. Results and Discussion

### 3.1. Sequence Analysis

**Figure 3:**
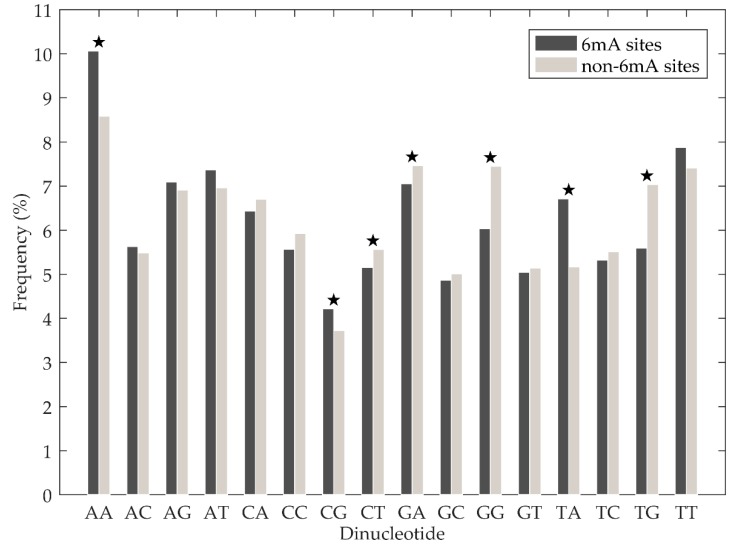
Averaged dinucleotide composition of positive and negative samples. The star indicates that there is statistically significant of difference on occurrence frequencies of corresponding dinucleotide between positive and negative samples.

Chen et al. [18] analysed the nucleotide composition difference between 6mA site-containing sequences and non-6mA site-containing sequences. They found that the adenosine and thymine nucleotides displayed significant enrichment in 6mA site containing sequences, while cytosine and guanine nucleotides were significantly enriched in non-6mA site containing sequences. In this study, we investigated the statistical significance of the dinucleotide composition difference between 6mA site-containing sequences and non-6mA sit- containing sequences further. First, normality tests were performed on the occurrence frequencies of 16 basic dinucleotide elements from positive and negative samples of training set using the Lilliefors test. We found that all the *p*-values were less than 0.05, which means those occurrence frequencies were not from normal distribution. Thus, we used the Mann–Whitney U-test to analyse the differences. As shown in Figure 3 (see Supplementary Table S3 for more details), there were statistically significant differences for occurrence frequencies of AA, CG, TA, CT, GA, GG, and TG between positive and negative samples, with the *p*-values less than 0.05. Furthermore, AA, CG, and TA dinucleotides were significantly enriched in 6mA site-containing sequences, while CT, GA, GG, and TG dinucleotides were significantly enriched in non-6mA site-containing sequences. The above results suggest that dinucleotide composition in a DNA sequence is important for discriminating between 6mA and non-6mA sites.

### 3.2. Performance Evaluation Using 10-Fold Cross-Validation Tests

According to the proposed feature extraction method described in Section 2.2, each DNA sequence was encoded into a 616-dimensional feature vector. Five classifiers mentioned in Section 2.4 were used to construct prediction models, which were then evaluated by identical 10 rounds of random 10-fold cross-validations. For SVM, the penalty parameter *C* (8) and kernel width *γ* (0.015625) were optimized by 10-fold cross-validation with grid search strategy in the search space {2^−5^, 2^−4^, …, 2^15^} and {2^−15^, 2^−14^, …, 2^5^}, respectively. For LogitBoost and TreeBagging, the number of basic learners was set to 100. The averaged prediction results are shown in Table 1. As can be seen, the performances of two ensemble classifiers (i.e., LogitBoost and TreeBagging) were better than those of individual classifiers. TreeBagging obtained the highest values in terms of Sp (>86%), Acc (>85%), MCC (>0.7), and AUC (>0.92) when compared with other four classifiers.

### 3.3. The Effect of Optimized DNA Properties on the Model Performance

To investigate whether the DNA property selection process can provide a positive effect on prediction performance and which properties are more competent to identify 6mA sites, we performed the property selection process described in Section 2.3. For each classifier, we first executed 10 rounds of random 10-fold partitions, and then implemented Algorithm 1 based on each 10-fold partition. For fairness, above 10 rounds of 10-fold partitions were identical for all the classifiers. The results are shown in Supplementary Tables S4–S8. As we expected, due to the randomness of each 10-fold partition, the selected properties and the corresponding accuracies differed in different rounds of 10-fold partitions, even if the same classifier was used. Meanwhile, it can be observed that some properties were frequently selected in different rounds. Taking the naive Bayes’ results as an example, properties 1, 8, 10, 11, and 13 appeared in all the property sets; property 12 was simultaneously selected in eight property sets.

**Figure 4:**
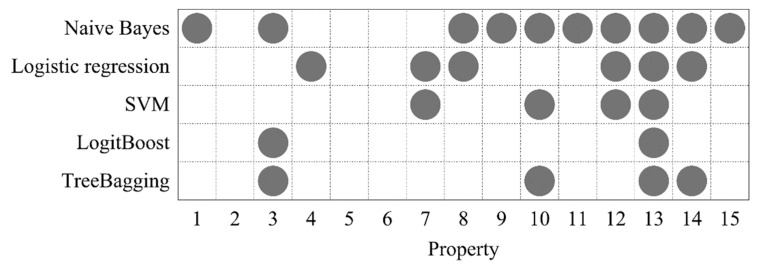
The optimized DNA properties for five classifiers.

In order to partially offset the randomness and further increase the reliability of the selected properties, we calculated the occurrence frequencies of each property among the 10 selected property sets, and then filtered out those with occurrence frequencies lower than threshold *λ* (0 ≤ *λ* ≤ 1). The larger *λ* means the remaining properties are more frequently selected. We think such properties are more reliable for identifying 6mA sites. For each classifier, the parameter *λ* was optimized by grid search in the search space [0, 1] with a step of 0.1, where the accuracies were calculated by averaging identical 10 rounds random 10-fold cross-validations, as above. As a result, the optimal *λ* values of 0.5, 0.5, 0.5, 0.5, and 0.6 were obtained for the five classifiers, respectively (Supplementary Table S9). This indicated that only the properties, which are selected with at least 50% probability in one round, can be considered as the members of the final optimized property set. The final optimized DNA properties for each classifier are shown in Figure 4. As can be seen, properties 3, 10, 12, 13, and 14 were selected at least three times, where property 13 appeared in all the selected property sets. This suggested that these properties display a more reliable ability to distinguish between 6mA and non-6mA sites. It is worth noting that property 3 (F-twist) and properties 10 (slide) and 12 (rise) are dinucleotide flexibility parameters and structure parameters which reflect DNA physical properties. Properties 13 (energy) and 14 (enthalpy) are DNA thermodynamic properties. It is self-evident as to why DNA physical properties and thermodynamic properties were used to generate DNA features in this study.

Table 2 lists the 10-fold cross-validation results based on optimized DNA properties for the five classifiers. In contrast with the predicted results based on all the 15 DNA properties (Table 1), various metrics were all improved to different degrees. For example, the accuracies were increased by 0.68%–1.44%. In addition, similar to the trend displayed in Table 1, TreeBagging still performed best among all the investigated classifiers. In view of the analysis above, it can be concluded that the proposed property selection method could output reliable and effective DNA property set to identify 6mA sites. TreeBagging and DNA features from the optimized properties 3 (F-twist), 10 (slide), 13 (energy) and 14 (enthalpy) were used together to build prediction model in the following.

### 3.4. Comparison with Other Methods

The number of base learners is an important parameter, which affects the performance of bagging algorithm. To optimize the number of decision trees, seven TreeBagging prediction models with 10, 50, 100, 200, 300, 400, and 500 decision trees were tested. The prediction model with 300 decision trees achieved the best results (Supplementary Table S10). Therefore, we used 300 decision trees to build the final prediction model, i6mA-DNCP, to identify 6mA sites.

To demonstrate the effectiveness of our method, we compared it with the recently proposed methods on the same dataset. The jackknife test results of these methods and i6mA-DNCP are summarized in Table 3. As can be seen, our method outperformed i6mA-Pred on all the metrics; the accuracy, MCC and AUC were improved by 3.52%, 0.074, and 0.04, respectively. Considering that i6mA-Pred is an SVM-based prediction model, we also used SVM and the corresponding optimized DNA features to build a prediction model for a more objective comparison. The jackknife test results of this model are also listed in Table 3. As can be seen, our SVM-based method still performed better than i6mA-Pred, with the accuracy, MCC, and AUC increased by 1.93%, 0.041, and 0.029, respectively. Compared with the recent method iDNA6mA-Rice, i6mA-DNCP outperformed it by 0.57% in terms of sensitivity, 5.45% in terms of specificity, 3.02% in terms of accuracy, 0.064 in terms of MCC, and 0.016 in terms of AUC. It worth noting that iDNA6mA-Rice is based on random forest, which is similar to TreeBagging used to construct our model. Therefore, it can be inferred that our features are more effective. In addition, i6mA-DNCP also reached the similar aggregate metrics of accuracy, MCC, and AUC to those obtained by iDNA6mA. We attribute the better performance to our effective DNA sequence-encoding scheme. That is, dinucleotide composition and dinucleotide-based DNA properties are more informative for identifying 6mA sites, in contrast with nucleotide composition, nucleotide properties, and the simple mono-nucleotide binary encoding. And we also noticed that our method performed best on specificity among all the compared methods but worse on sensitivity than iDNA6mA and our SVM-based method. Thus, these methods show complementarity. To improve performance further, integrating different kinds of feature representations and classifiers to generate an ensemble classifier-based model will be an effective strategy.

As a widely used feature model in computational genomics, PseDNC [37,38,39], incorporates dinucleotide composition and six DNA physical properties (i.e. shift, slide, rise, twist, tilt, and roll) into dimension-fixed feature vectors to reflect both the local and global sequence-pattern information of genomic sequences. The jackknife test results of the PseDNC-based method are reported in Table 3. Although the dinucleotide composition and similar DNA properties were also used in this study, our method performed much better than the PseDNC-based method. The accuracy of the PseDNC-based method was 22.1% lower than that of i6mA-DNCP. An explanation may be that, in contrast to integrating dinucleotide composition and DNA properties by correlation transformation, which is performed in PseDNC, it would be more effective at using dinucleotide composition and DNA property parameters directly to identify 6mA sites.

### 3.5. Validation on Independent Datasets

To demonstrate whether our method could recognize the 6mA sites in other species, we validated i6mA-DNCP by performing the following independent dataset tests. Following similar procedures as described those in [18], we constructed three datasets of 6mA sites-containing sequences from the genomes of *Arabidopsis thaliana*, *Fragaria vesca*, and *Rosa chinensis*. For the first dataset, 6mA site-containing sequences were extracted from NCBI Gene Expression Omnibus (GEO) with accession number GSE81597 [13]. A total of 189,587 sequences were obtained. For the latter two datasets, a total of 26,514 and 14,677 6mA site-containing sequences for *Fragaria vesca* and *Rosa chinensis* genomes were obtained from the MDR database [40]. Then, only the sites with modification scores of at least 30 were reserved. After high homologous sequences (with more than 80% similarity) were removed using CD-HIT web-server [41] (http://weizhongli-lab.org/cd-hit/), we obtained the final independent datasets with 27,751, 8983 and 1479 samples for *Arabidopsis thaliana*, *Fragaria vesca*, and *Rosa chinensis* genomes, respectively.

The predicted results on the independent dataset tests are listed in Table 4. We found that the success rates obtained by using the model trained by the benchmark dataset from the rice genome to the genomes of other three organisms were all very high. It indicates that i6mA-DNCP is indeed quite promising and holds a high potential to become a useful tool in genome-wide analysis for identifying 6mA sites.

## 4. Conclusions

In view of the significance of 6mA in regulating gene expression in eukaryotes, it is meaningful to develop high-quality computational model for identifying 6mA sites to facilitate characterizing genomic 6mA distributions and other downstream studies. By encoding the DNA samples using dinucleotide composition and optimized dinucleotide-based DNA properties, a new prediction method named i6mA-DNCP was proposed in the current study. According to the jackknife evaluation, i6mA-DNCP outperformed the state-of-the-art methods. We attribute the success of i6mA-DNCP to three factors: (1) straightforwardness, but an informative, dinucleotide-based DNA sequence-encoding method; (2) a reliable DNA property selection strategy to optimize feature space; and (3) a powerful bagging classifier to effectively utilize the extracted DNA features. It is anticipated that i6mA-DNCP will become an essential computational tool for identifying 6mA sites in the rice genomes. The codes are publicly available at https://ww2.mathworks.cn/matlabcentral/fileexchange/72549-i6mA-dncp.

## Figures and Tables

**Table 1:** Performance comparison of different classifiers by 10-fold cross-validations based on 15 DNA properties.

Classifier	Sn (%)	Sp (%)	Acc (%)	MCC	AUC
Naive Bayes	81.02	79.63	80.32	0.607	0.879
Logistic regression	82.23	80.76	81.49	0.630	0.897
SVM	84.60	82.89	83.74	0.675	0.914
LogitBoost	84.16	84.77	84.47	0.689	0.916
TreeBagging	84.32	86.36	85.34	0.707	0.921

**Table 2:** Performance comparison of different classifiers by 10-fold cross-validations based on optimized properties.

Classifier	Sn (%)	Sp (%)	Acc (%)	MCC	AUC
Naive Bayes	82.67	80.84	81.76	0.635	0.889
Logistic regression	83.42	81.85	82.64	0.653	0.901
SVM	85.47	83.61	84.54	0.691	0.915
LogitBoost	84.86	85.43	85.15	0.703	0.917
TreeBagging	84.09	88.07	86.08	0.722	0.926

**Table 3:** Performance comparison of different methods by the jackknife test.

Method	Sn (%)	Sp (%)	Acc (%)	MCC	AUC
i6mA-Pred	82.95	83.30	83.13	0.660	0.886
PseDNC	63.52	65.57	64.55	0.290	0.636
iDNA6mA	86.70	86.59	86.64	0.730	0.931
iDNA6mA-Rice	83.86	83.41	83.63	0.670	0.910
i6mA-DNCP	84.43	88.86	86.65	0.734	0.926
SVM-based method	86.25	83.86	85.06	0.701	0.915

The prediction results for i6mA-Pred, PseDNC, iDNA6mA, and iDNA6mA-Rice are taken from [18,19,20], respectively. The “SVM-based method” denotes the method based on SVM and the corresponding optimized DNA features in this study. The penalty parameter *C* and kernel width *γ* are optimized as 2 and 0.03125, respectively.

**Table 4:** Predicted results by i6mA-DNCP on the samples collected from three other genomes.

Genome	Number of Samples	Number of Corrected Prediction	Success Rate (%)
*Arabidopsis thaliana*	27,751	25,394	91.51
*Fragaria vesca*	8983	8680	96.63
*Rosa chinensis*	1479	1359	91.89

## Supplementary Materials

The following are available online at https://www.mdpi.com/2073-4425/10/10/828/s1: Table S1: The original dinucleotide property; Table S2: The normalized dinucleotide property corresponding to Table S1; Table S3: The statistical significance of the dinucleotide composition difference between 6mA site containing sequences and non-6mA site containing sequences; Table S4: The selected DNA properties and the corresponding accuracies in 10 rounds of implementations of Algorithm 1 based on Naive Bayes; Table S5: The selected DNA properties and the corresponding accuracies in 10 rounds of implementations of Algorithm 1 based on logistic regression; Table S6: The selected DNA properties and the corresponding accuracies in 10 rounds of implementations of Algorithm 1 based on SVM; Table S7: The selected DNA properties and the corresponding accuracies in 10 rounds of implementations of Algorithm 1 based on LogitBoost; Tabel S8: The selected DNA properties and the corresponding accuracies in 10 rounds of implementations of Algorithm 1 based on TreeBagging; Table S9: Averaged accuracies (%) corresponding to varying *λ* for five classifiers; Table S10: Accuracies corresponding to different numbers of base learners.
