# Supplementary material for: i6mA-DNCP: Computational Identification of DNA N6-Methyladenine Sites in the Rice Genome Using Optimized Dinucleotide-Based Features
Source: Genes (Basel). 2019 Oct 20;10(10):828. doi: 10.3390/genes10100828 (PMC6826501; doi:10.3390/genes10100828)
Supplement: Supplementary file 1 [file genes-10-00828-s001.zip › genes-593012-SI.docx]

Table S1. The original dinucleotide property.

| Property index | AA/TT | AT | AG/CT | AC/GT | TA | TG/CA | TC/GA | GG/CC | GC | CG |
| --- | --- | --- | --- | --- | --- | --- | --- | --- | --- | --- |
| F-roll | 0.041 | 0.054 | 0.042 | 0.065 | 0.031 | 0.035 | 0.049 | 0.041 | 0.054 | 0.039 |
| F-tilt | 0.078 | 0.098 | 0.058 | 0.071 | 0.065 | 0.056 | 0.065 | 0.057 | 0.065 | 0.059 |
| F-twist | 0.069 | 0.071 | 0.053 | 0.064 | 0.048 | 0.052 | 0.057 | 0.055 | 0.059 | 0.051 |
| F-slide | 6.689 | 9.611 | 3.472 | 6.803 | 1.853 | 2.003 | 4.268 | 2.99 | 4.206 | 2.713 |
| F-shift | 6.239 | 4.658 | 2.801 | 2.911 | 4.107 | 2.882 | 3.58 | 2.67 | 2.655 | 3.019 |
| F-rise | 21.34 | 24.792 | 17.477 | 21.977 | 14.239 | 14.512 | 18.41 | 14.252 | 17.311 | 14.655 |
| Roll | 1.051 | 0.612 | 3.6 | 2.005 | 3.499 | 5.6 | 2.444 | 4.682 | 1.697 | 6.015 |
| Tilt | −1.261 | 0 | −1.655 | 0.334 | 0 | 0.137 | 1.437 | −0.77 | 0 | 0 |
| Twist | 35.02 | 30.72 | 32.29 | 31.53 | 36.94 | 35.43 | 35.67 | 33.54 | 34.07 | 33.67 |
| Slide | −0.176 | −0.679 | −0.223 | −0.593 | 0.044 | 0.481 | −0.046 | −0.166 | −0.19 | 0.443 |
| Shift | 0.013 | 0 | −0.023 | −0.018 | 0 | 0.009 | −0.011 | 0.026 | 0 | 0 |
| Rise | 3.253 | 3.208 | 3.322 | 3.243 | 3.389 | 3.366 | 3.299 | 3.361 | 3.267 | 3.291 |
| Energy | −1 | −0.88 | −1.28 | −1.44 | −0.58 | −1.45 | −1.3 | −1.84 | −2.24 | −2.17 |
| Enthalpy | −7.6 | −7.2 | −7.8 | −8.4 | −7.2 | −8.5 | −8.2 | −8 | −9.8 | −10.6 |
| Entropy | −21.3 | −20.4 | −21 | −22.4 | −21.3 | −22.7 | −22.2 | −19.9 | −24.4 | −27.2 |

The properties in the first 6 lines denote dinucleotide flexibility parameters. The second 6 lines denote dinucleotide structure parameters, and the bottom 3 lines denote thermodynamic parameters.

Table S2. The normalized dinucleotide property corresponding to Table S1.

| Property index | AA/TT | AT | AG/CT | AC/GT | TA | TG/CA | TC/GA | GG/CC | GC | CG |
| --- | --- | --- | --- | --- | --- | --- | --- | --- | --- | --- |
| F-roll | −0.438 | 0.902 | −0.335 | 2.035 | −1.468 | −1.056 | 0.386 | −0.438 | 0.902 | −0.644 |
| F-tilt | 1.094 | 2.927 | −0.739 | 0.453 | −0.097 | −0.922 | −0.097 | −0.831 | −0.097 | −0.647 |
| F-twist | 1.572 | 1.859 | −0.727 | 0.853 | −1.446 | −0.871 | −0.153 | −0.440 | 0.135 | −1.015 |
| F-slide | 1.039 | 2.382 | −0.439 | 1.092 | −1.183 | −1.114 | −0.073 | −0.660 | −0.102 | −0.788 |
| F-shift | 2.332 | 0.967 | −0.636 | −0.541 | 0.491 | −0.566 | 0.036 | −0.749 | −0.762 | −0.448 |
| F-rise | 1.018 | 2.050 | −0.136 | 1.209 | −1.104 | −1.023 | 0.142 | −1.100 | −0.186 | −0.980 |
| Roll | −1.239 | −1.497 | 0.257 | −0.679 | 0.198 | 1.432 | −0.421 | 0.893 | −0.860 | 1.675 |
| Tilt | −1.133 | 0.242 | −1.563 | 0.607 | 0.242 | 0.392 | 1.810 | −0.598 | 0.242 | 0.242 |
| Twist | 0.638 | −1.805 | −0.913 | −1.345 | 1.729 | 0.871 | 1.007 | −0.203 | 0.098 | −0.129 |
| Slide | −0.180 | −1.651 | −0.318 | −1.399 | 0.462 | 1.740 | 0.199 | −0.151 | −0.221 | 1.629 |
| Shift | 0.876 | 0.032 | −1.461 | −1.136 | 0.032 | 0.617 | −0.682 | 1.720 | 0.032 | 0.032 |
| Rise | −0.932 | −1.776 | 0.362 | −1.120 | 1.619 | 1.188 | −0.069 | 1.094 | −0.669 | −0.219 |
| Energy | 0.936 | 1.213 | 0.290 | −0.079 | 1.905 | −0.102 | 0.244 | −1.002 | −1.925 | −1.764 |
| Enthalpy | 0.746 | 1.214 | 0.512 | −0.190 | 1.214 | −0.307 | 0.044 | 0.278 | −1.829 | −2.765 |
| Entropy | 0.411 | 0.925 | 0.582 | −0.218 | 0.411 | −0.389 | −0.104 | 1.210 | −1.360 | −2.960 |

Table S3. The statistical significance of the dinucleotide composition difference between 6mA site containing sequences and non-6mA site containing sequences.

|  | AA | AC | AG | AT | CA | CC | CG | CT | GA | GC | GG | GT | TA | TC | TG | TT |
| --- | --- | --- | --- | --- | --- | --- | --- | --- | --- | --- | --- | --- | --- | --- | --- | --- |
| Averaged occurrence frequency in 6mA sites | 0.101 | 0.056 | 0.071 | 0.074 | 0.064 | 0.056 | 0.042 | 0.052 | 0.071 | 0.049 | 0.060 | 0.050 | 0.067 | 0.053 | 0.056 | 0.079 |
| Averaged occurrence frequency in non-6mA sites | 0.086 | 0.055 | 0.069 | 0.070 | 0.067 | 0.059 | 0.037 | 0.056 | 0.075 | 0.050 | 0.074 | 0.051 | 0.052 | 0.055 | 0.070 | 0.074 |
| Mann-Whitney U-test *p*-value | **0.001** | 0.378 | 0.757 | 0.071 | 0.219 | 0.088 | **0.008** | **0.008** | **0.019** | 0.106 | **0.000** | 0.680 | **0.000** | 0.162 | **0.000** | 0.410 |

The *p*-values less than 0.05 are highlighted in bold face.

Table S4. The selected DNA properties and the corresponding accuracies in 10 rounds of implementations of Algorithm 1 based on Naive Bayes.

| Round | Acc (%) | Serial number of selected properties |
| --- | --- | --- |
| 1 | 82.33 | 1, 8, 10, 11, 12, 13, 14 |
| 2 | 82.16 | 1, 3, 8, 9, 10, 11, 12, 13, 14, 15 |
| 3 | 82.44 | 1, 3, 8, 9, 10, 11, 12, 13, 14 |
| 4 | 81.99 | 1, 7, 8, 9, 10, 11, 12, 13, 15 |
| 5 | 82.33 | 1, 3, 8, 10, 11, 13, 14 |
| 6 | 82.84 | 1, 8, 9, 10, 11, 12, 13, 15 |
| 7 | 82.44 | 1, 7, 8, 9, 10, 11, 12, 13, 14 |
| 8 | 82.27 | 1, 3, 8, 9, 10, 11, 12, 13, 15 |
| 9 | 82.39 | 1, 3, 8, 10, 11, 13, 15 |
| 10 | 82.67 | 1, 7, 8, 9, 10, 11, 12, 13, 14 |

Table S5. The selected DNA properties and the corresponding accuracies in 10 rounds of implementations of Algorithm 1 based on logistic regression.

| Round | Acc (%) | Serial number of selected properties |
| --- | --- | --- |
| 1 | 82.95 | 4, 6, 7, 8, 12, 13, 14 |
| 2 | 83.13 | 4, 7, 13 |
| 3 | 83.52 | 12, 13, 14 |
| 4 | 82.84 | 4, 10, 12, 13, 15 |
| 5 | 83.75 | 7, 8, 13, 14, 15 |
| 6 | 82.95 | 4, 7, 10, 13 |
| 7 | 83.75 | 7, 8, 13, 14 |
| 8 | 82.50 | 4, 6, 8, 10, 13, 14, 15 |
| 9 | 83.30 | 4, 5, 6, 7, 9, 12, 13 |
| 10 | 83.30 | 2, 4, 8, 9, 10, 12, 13, 14 |

Table S6. The selected DNA properties and the corresponding accuracies in 10 rounds of implementations of Algorithm 1 based on SVM.

| Round | Acc (%) | Serial number of selected properties |
| --- | --- | --- |
| 1 | 85.00 | 6, 7, 9, 10, 12, 13 |
| 2 | 85.40 | 7, 10, 13 |
| 3 | 85.06 | 7, 10, 13 |
| 4 | 85.23 | 6, 10, 12, 13 |
| 5 | 85.40 | 10, 12, 13, 14 |
| 6 | 84.94 | 3, 10, 12, 14 |
| 7 | 84.55 | 7, 10, 12, 13 |
| 8 | 85.51 | 5, 7, 10, 13 |
| 9 | 84.83 | 3, 10, 11, 13 |
| 10 | 84.72 | 6, 8, 10, 13, 14 |

Table S7. The selected DNA properties and the corresponding accuracies in 10 rounds of implementations of Algorithm 1 based on LogitBoost.

| Round | Acc (%) | Serial number of selected properties |
| --- | --- | --- |
| 1 | 85.80 | 3, 6, 13 |
| 2 | 85.91 | 5, 7, 9, 12, 13, 14 |
| 3 | 85.57 | 2, 3, 13 |
| 4 | 85.68 | 3, 5, 7 |
| 5 | 85.74 | 2, 3, 6, 14 |
| 6 | 85.40 | 3, 5, 6, 7, 8, 10, 12 |
| 7 | 85.57 | 2, 3, 5, 14 |
| 8 | 85.45 | 2, 3, 13, 14 |
| 9 | 85.80 | 3, 13 |
| 10 | 85.68 | 3, 13 |

Table S8. The selected DNA properties and the corresponding accuracies in 10 rounds of implementations of Algorithm 1 based on TreeBagging.

| Round | Acc (%) | Serial number of selected properties |
| --- | --- | --- |
| 1 | 86.88 | 2, 3, 5, 7, 10, 13, 14, 15 |
| 2 | 86.65 | 2, 3, 6, 10, 13, 14 |
| 3 | 86.70 | 2, 3, 5, 10, 13, 14, 15 |
| 4 | 86.25 | 3, 4, 6, 7, 8, 13, 14 |
| 5 | 86.19 | 2, 3, 5, 7, 8, 10, 11, 12, 13, 15 |
| 6 | 86.08 | 3, 5, 7, 9, 10, 11, 13, 15 |
| 7 | 86.08 | 3, 6, 10, 12, 13, 15 |
| 8 | 86.48 | 3, 5, 7, 8, 10, 13, 14, 15 |
| 9 | 86.31 | 3, 10, 11, 12, 13, 14 |
| 10 | 86.19 | 3, 5, 14 |

Table S9. Averaged accuracies (%) corresponding to varying *λ* for five classifiers.

| *λ* | Naive Bayes | Logistic regression | SVM | LogitBoost | TreeBagging |
| --- | --- | --- | --- | --- | --- |
| 0 | 80.32 | 81.49 | 83.74 | 84.47 | 85.34 |
| 0.1 | 81.39 | 81.64 | 83.48 | 84.47 | 85.45 |
| 0.2 | 81.39 | 82.17 | 83.51 | 84.79 | 85.85 |
| 0.3 | 81.76 | 82.34 | 84.54 | 84.93 | 86.14 |
| 0.4 | 81.76 | 82.34 | 84.54 | 84.93 | 85.86 |
| 0.5 | **81.76** | **82.64** | **84.54** | **85.15** | 85.85 |
| 0.6 | 80.90 | 81.32 | 82.56 | 84.16 | **86.31** |
| 0.7 | 81.37 | 73.67 | 82.56 | 84.16 | 85.45 |
| 0.8 | 81.37 | 73.67 | 82.56 | 84.16 | 85.57 |
| 0.9 | 81.15 | 73.67 | 82.56 | 84.16 | 85.28 |

The best results are highlighted in bold face. When there are identical accuracies with different *λ*, the maximum *λ* is fetched.

Table S10. Accuracies corresponding to different numbers of base learners.

| Number of base learners | 10 | 50 | 100 | 200 | 300 | 400 | 500 |
| --- | --- | --- | --- | --- | --- | --- | --- |
| Accuracy (%) | 80.63 | 84.03 | 86.08 | 86.25 | **86.53** | 86.42 | 86.36 |

The best results are highlighted in bold face.
